# Parallel alternating sliding knots are effective for ligation of mesenteric arteries during resection and anastomosis of the equine jejunum

**DOI:** 10.1186/1746-6148-10-S1-S10

**Published:** 2014-07-07

**Authors:** Marco Gandini, Gessica Giusto, Francesco Comino, Eleonora Pagliara

**Affiliations:** 1 Department of Veterinary Sciences, University of Turin, Via L. da Vinci 44, Grugliasco (TO), Italy

## Abstract

**Background:**

In literature only one article describes and compares methods of achieving hemostasis in equine mesenteric arteries during jejunal resection and anastomosis, and most textbooks favor ligating-dividing mechanical devices. The latter method cannot always be used, not least because the devices are expensive and in some cases even contra-indicated. Various types of knots, including sliding knots, are widely used to provide hemostasis in laparoscopy.

The objective of this study was to compare a triple ligature for mesenteric vessels composed of three sliding knots with a triple ligature composed of a modified transfixing and two surgeon’s knots.

**Methods:**

Portions of jejunum with associated mesenteric vessels were collected from 12 horses at a local abattoir. These were divided into 24 specimens containing five mesenteric arteries each. Each artery was closed with a triple ligature. In group A, a surgeon’s knot was used to tie the ligatures (two circumferential and one modified transfixing) while in group B all ligatures (three circumferential) were tied with a parallel alternating sliding knot. Both groups were divided ino two subgroups depending on suture material used (multifilament or monofilament suture material). Time to perform ligatures for every specimen were recorded and compared between groups.

After closure, arteries were cannulated and intraluminal pressures were increased until ligature failure. Leaking pressures were recorded and compared between groups.

**Results:**

Ligation of mesenteric arteries was significantly faster to perform with sliding knots than with surgeon’s knots, both with monofilament and multifilament suture material. With multifilament suture material, the leaking pressure of sliding knot ligatures was significantly higher than that of surgeon’s knot ligatures. With monofilament suture, there were no statistically significant differences in leaking pressure between ligature methods. Both ligating methods were stronger with monofilament suture material than with multifilament suture material.

**Conclusions:**

Regardless of the ligature used, monofilament suture material performed better than multifilament suture material to achieve hemostatic knots. Independently of the suture material, the sliding knot is comparable or better than the surgeon’s knot in providing hemostasis, and is faster to perform.

## Background

Intestinal resection and anastomosis are techniques commonly performed in equine abdominal surgery[1]. These procedures necessitate ligation of mesenteric or intestinal vessels, sometimes in large numbers in a single patient. Extensive small intestinal resection and anastomosis may require ligating and transecting up to 15 or 20 arteries which necessitates considerable surgical time.

In the existing literature we could find only one article in which three methods of providing hemostasis of mesenteric arteries in horses are compared [2]. In most standard textbooks [1,3-6] only one method, based on a ligating-dividing mechanical device, is described, and only two describe ligation as an alternative method of haemostasis of the mesenteric vessels [3,7]. In our experience, use of ligating-dividing mechanical devices cannot always be employed, not least because they are expensive, but also because if the mesentery is edematous or the vessels dilated, the use of these devices is not recommended and the use of hemostatic ligatures is required [3]. In addition, within equine surgical textbooks there is a lack of description of general purpose hemostatic ligatures [1,3-6]. In the article by Rumbaugh *et al*[2], the ligating-dividing stapler was compared with an energy-based vessel sealing device and with a double ligature including a circumferential and a modified transfixing ligature. Both ligatures used a surgeon’s knot to tie the suture, although this knot is considered unreliable for hemostatic ligatures [8].Sliding knots are widely used in equine laparoscopy and recently have been described as hemostatic knots in other species [8-10]. They are quick and easy to perform and behave as or better than the surgeon’s knot when used to provide hemostasis of arteries [8].

The purpose of this study was to compare two ligatures, performed with mono- and multifilament suture material, for providing hemostasis during small intestinal resection and anastomosis in horses. To mimic the clinical setting, we compared the leaking pressure and construction time of a triple ligature composed of three sliding knots with a triple ligature composed of a modified transfixing and two surgeon’s knots.

## Methods

Twenty four portions of jejunum complete with mesentery and mesenteric vessels were used for the study. Specimens were harvested immediately after death from12 healthy slaughtered horses (mean age 26 months, range 18-30 months, mean weight 450 kg, range 420-480) at the Didactical Abattoir, Department of Veterinary Sciences, University of Turin, washed, cleaned and stored in warm 0.9% sodium chloride solution. The experiments were performed within 6 hours following collection. Each specimen included a length of intestine with five associated mesenteric arteries.

**Figure 1:**
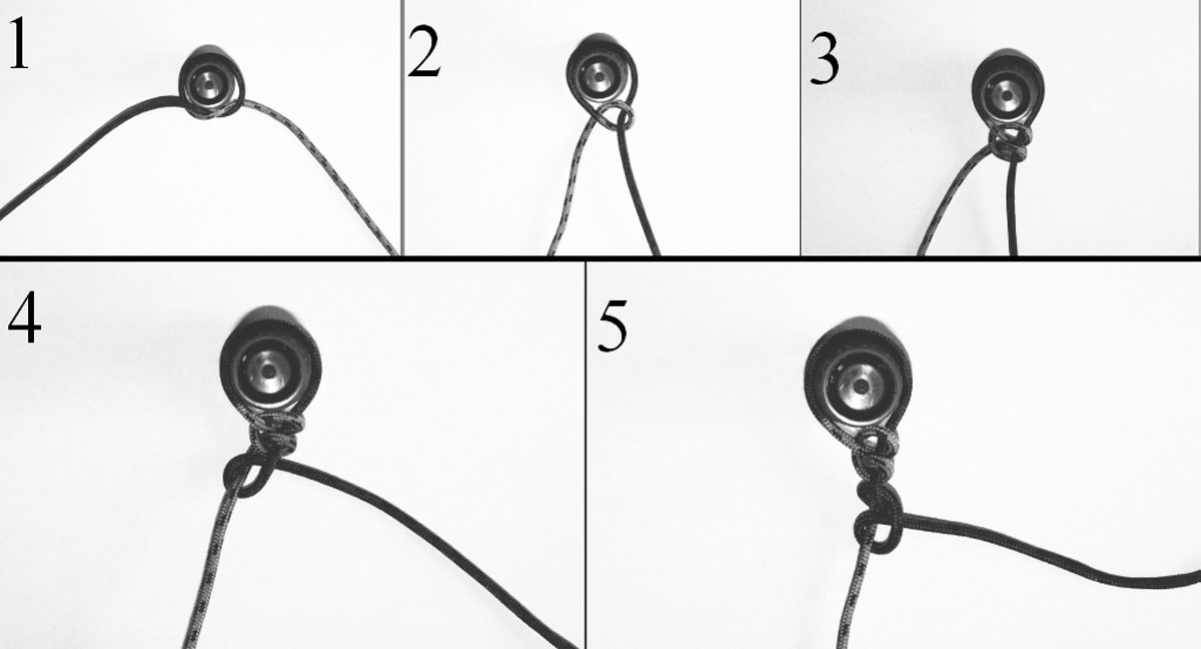
Steps to tie a parallel sliding knot with alternating post 1: a simple knot is tied around the vessel 2: by pulling on the yellow strand the knot is reversed in a slip knot around the blue strand that from now on act as “post” 3: another loop is formed around the post forming a “half-hitch knot” also called “parallel sliding knot” 4: the procedure is then repeated, but now using the yellow strand as a post (alternating the post) 5: the completed knot : parallel sliding knot on alternating post.

Specimens were divided into two groups of 12 specimens each: in Group A, each mesenteric artery was ligated with three ligatures consisting of a circumferential ligature [1] proximally, followed by a transfixing ligature on the vascular bundle (mesenteric artery and vein) and a further circumferential ligature distally [2]; all ligatures were closed with a surgeon’s knot with four overthrows for a total of six throws [11]. In Group B, each artery was ligated with three circumferential ligatures tied with a sliding knot with two overthrows. The sliding knot we choose was a parallel sliding knot (half hitch ) because of its demonstrated superiority in holding strength compared to other sliding knots [12] with two overthrows on the opposite strand [13](Fig. 1). The two groups were further divided into two subgroups (6 specimens each) M and P. In the subgroup P, ligatures were tied using multifilament material (Lactomer 9-1), and in the subgroup M using monofilament (Glyconate) suture material, size 2-0 USP [1-3,7]. To avoid operator influence, all knots were performed by the same, experienced surgeon (MG). For each work session, four specimens were tested, to avoid surgeon’s fatigue. To mimic the clinical setting, arteries were dissected from the mesentery [7] and the surgeon was helped by an assistant; after completion the excess suture thread of each knot was cut to a standard length of 3 mm [14]. The tensile force applied by surgeon for creating the knot was not measured.

### Intestinal length

To better compare our results with parameters used in clinical practice to quantify the extent of a jejunal resection (that is the amount of intestine resected), the length of each specimen was measured.

For each specimen, the length in centimeters was measured on the antimesenteric border of the intestine, and then compared between groups and subgroups.

### Construction time

Construction time was recorded and compared between groups; time was measured starting when the needle was inserted through the mesentery to perform the first knot and it was stopped after the assistant cut the last knot’s thread.

### Leaking pressure

**Figure 2:**
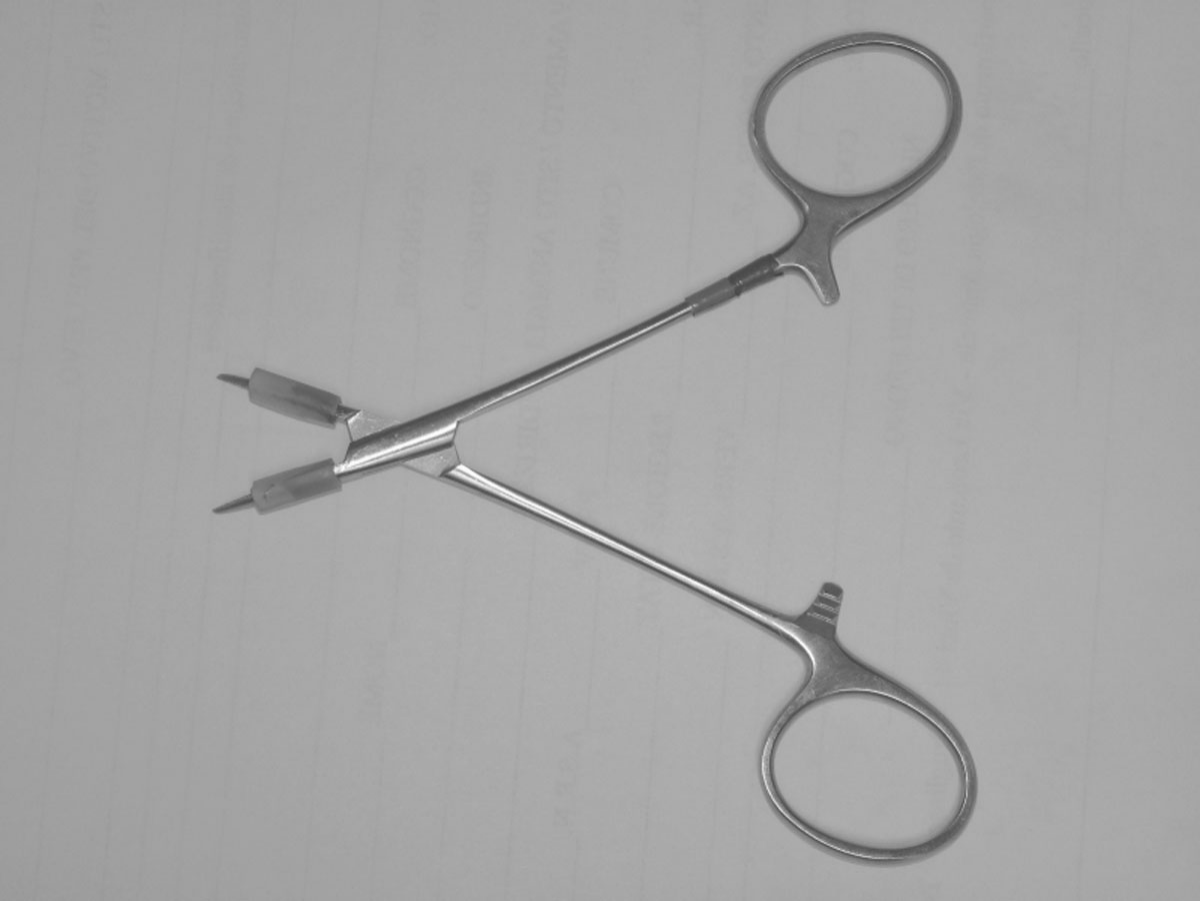
Two pieces of 5 mm latex tubing were placed around the jaws of a mosquito forceps.

**Figure 3:**
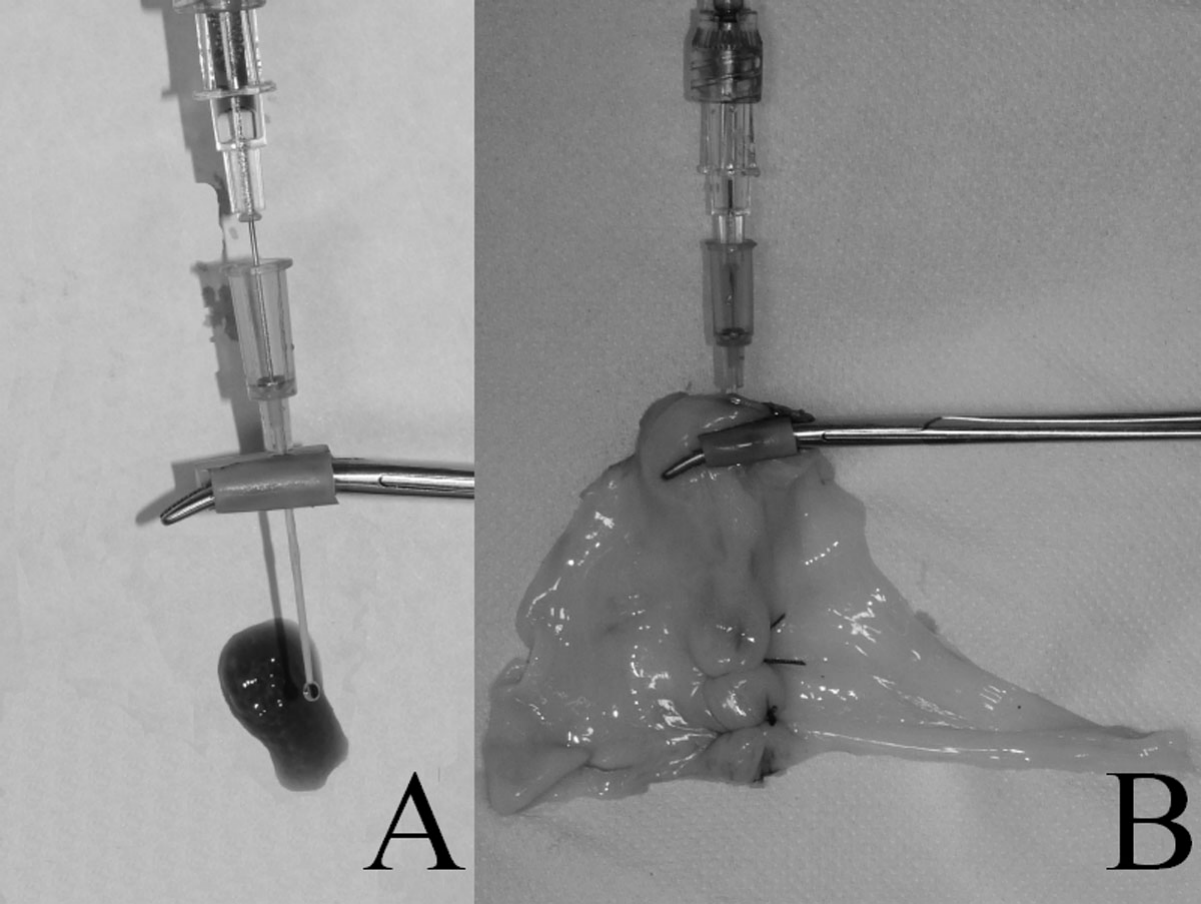
A) The intravenous catheter partially fed along the internal trocar and clamped with the mosquito forceps- note free flowing of fluid through the clamped catheter and trocar. B) The system inserted into the ligated mesenteric artery.

After performing each ligature, each mesenteric artery was transected between the second and third ligature (in a proximal-distal order). The artery was cannulated proximally with a 22G 1-1/4” intravenous catheter (Protective®Plus) about 5 mm from the proximal knot. The catheter was connected through a three-way stopcock to an analog pressure gauge and a 50 ml syringe using a technique similar to a previously published study [15]. Briefly two pieces of 5 mm latex tubing were placed around the jaws of a mosquito forceps (Fig. 2). The intravenous catheter was partially fed along the internal trocar, so to protect tissue from its sharp tip (Fig. 3A). The catheter was then introduced into the artery that was then clamped with the modified mosquito forceps (Fig. 3B). A solution of methylene blue dye and water was injected through the catheter until ligature failure, as identified by loss in pressure or dye leakage [2,7,15]; maximal luminal pressure was recorded and compared between groups. When pressures reached 1050 mmHg, injection was stopped. The leaking pressure test was performed by two operators blinded to the specimens’ group.

### Statistical analysis

Normal distribution of data was evaluated with the Kolmogorov and Smirnov test.

If data were normally distributed an *unpaired T test* was used. If data were not normally distributed the *Mann-Whitney test* was used. All statistical analyses were performed using statistical software (GrafpadInstat^®^, La Jolla, CA) with significance set to p<0.05.

## Results

### Bowel length

The unpaired T test was used to compare intestinal length of specimens. The bowel length of specimens in Group B was of 3.84 ± 0.53 m (mean ± SD) and 3.72 ±0.87 m for Group A. The difference was not statistically significant (p=0.1678). The bowel specimens’ length used to test multifilament suture material (subgroups A-P and B-P) was 3.98 ± 0.74 m (mean± SD), and 3.95 ± 0.81 m for monofilament suture material (subgroups A-M and B-M).This difference was also not statistically significant (p=0.9137).

### Construction time

The construction times were compared with the unpaired T-test. Construction time (mean±SD) was 6.39±0.13 minutes for the monofilament sliding knot (subgroup B-M) and 7.82±1.11 minutes for surgeon’s knot (group A-M). This difference was statistically significant (p=0.0120). For ligatures with multifilament suture material, construction time (mean±SD) was 7.12±0.86 minutes for sliding knots (subgroup B-P) and 8.46±0.57 minutes for surgeon’s knots (subgroup A-P). This difference was statistically significant (p=0.0102). There were no statistically significant differences between surgeon’s knots performed with monofilament (subgroup A-M, 7.82 ± 1.11 minutes) or multifilament suture material (subgroup A-P, 8.46 ± 0.57 minutes) (p=0.2191). The time to construct sliding knots with monofilament (subgroup B-M, 6.39 ± 0.13 minutes) and multifilament suture material (subgroup B-P, 7.12 ± 0.86 minutes) were not significantly different (p=0.2972).

### Leaking pressure

There was no rupture or leakage from the arteries due to ligature tying or pressure testing. All the pressure tests produced a leak of coloured fluid on the side of the artery opposite to catheter insertion site. Pressure data were not normally distributed, thus the leaking pressures were compared with the Mann-Whitney test. Leaking pressure for ligatures tied with monofilament suture material was 1050.0 mmHg (750-1050) for surgeon’s knots (Subgroup A-M) and 1050.00 (750-1050) mmHg for sliding knots (Subgroup B-M). This difference was not statistically significant (p=0.6282). Leaking pressure for ligatures performed with multifilament suture material was 468.75 mmHg (150-1050) for surgeon’s knots (subgroup A-P) and 1018.8 mmHg (300-1050) for sliding knots (subgroup B-P). The difference was statistically significant (p=0.0022). In group A (surgeon’s knot), there was a statistically significant difference (p< 0.001) in leaking pressure between ligatures created with monofilament [1050 mmHg (750-1050)] compared to multifilament suture material [468.75 mmHg (150-1050)]. In the group B (sliding knot) there was a statistically significant difference (p=0.0137) in leaking pressure between monofilament [(1050 mmHg (750-1050)] and multifilament suture material [1018.8 mmHg (300-1050)].

## Discussion

This *in vitro* study compared double ligatures with a circumferential and a transfixing suture with double ligatures with two sliding knots for mesenteric vessel closure during small intestinal resection in horses. In our study, sliding knots were as effective as surgeon’s knots in providing hemostasis in equine mesenteric arteries, but faster to perform. Both ligation methods were stronger with monofilament suture material than with multifilament suture material.

With our model, all sutures came into contact with the mesentery and its moistened surface, thus recreating the wet environment typical of abdominal surgery in horses that can impair suture knot holding ability [16]. With multifilament suture material, sliding knots resisted higher pressures than surgeon’s knots. While the sliding knots resisted intraluminal pressure well above those recorded in physiological and pathological circumstances, in some cases the intraluminal pressure that caused leakage with the surgeon’s knot was close to pressures recorded in live horses [17]. Furthermore, under general anaesthesia, blood pressure is generally lower than during the recovery. Thus knots applied when the animal is under anaesthesia may initially provide hemostasis, but may not resist higher pressures experienced during the recovery phases [18]. For this reason it is of paramount importance to choose a knot and suture material that can provide resistance to these higher pressures.

With the monofilament suture material, the two different ligating methods produced similar results and were more resistant to pressure than the surgeon’s knot technique performed with multifilament suture material. This difference is likely due to the elastic properties of the monofilament suture that give more grip to the stretched portion within the knots as previously described[19]. Recent studies on several knot types using monofilament suture material demonstrated that under tension the square knot changes its conformation into a sliding knot [19]. This alteration generates stress on the thread that breaks precisely at the point where knot’s configuration mutates [20,21]. Thus it is probably more advantageous with a monofilament suture material to perform a sliding knot from the beginning since its vessel sealing properties are equal to or better than a transfixing ligature performed using a surgeon’s knot.

Surgery time is an important factor in equine abdominal surgery [22]. Sliding knots were generally faster to tie than surgeon’s knots, both with multifilament and monofilament suture material. In extensive small intestinal resection, when considerable length of intestine is removed, this may account for a saving of approximately 10 minutes. Although this time saving alone may not be crucial for the outcome of the surgery it could contribute to a reduced surgical time.

It is difficult to compare our results with the existing literature because of a lack of details in other studies. The only comparable results to ours are the pressures resisted by transfixing ligatures with monofilament suture material reported by Rumbaugh and colleagues [2].

Our results demonstrate that in the case of an extensive small bowel resection if LDS or Ligasure devices are not available, or if the surgeon prefers to use these devices with the addition of a ligature for better hemostatic efficacy [7,18], sliding knots may save time as well as providing good hemostasis. Furthermore in cases where the surgeon decides to collect the mesentery with a first knot’s thread [3], the sliding knot provides a better choice because the mesentery weight results in self-lockage of the sliding knot. The use of monofilament suture material is recommended because it appears to provide better hemostasis regardless of which knot is chosen, and because of its known knot holding ability.

Limitations of our work include the fact that the study was conducted on healthy animals whereas mesenteric arteries in horses requiring intestinal resection and anastomosis may have undergone stresses that could diminish the hemostatic properties of the ligatures, for example tearing of the arterial wall when tying the knot. Another limitation is the fact that the operator that performed the ligatures was obviously not blinded, and this could lead to potential bias in the construction time.

Our study was limited to *in vitro* testing, and we cannot rule out the possibility that ligatures could behave differently when applied to live animals in pathological conditions. A further limitation of our work is that we didn’t consider tension applied by the surgeon. To mimic a clinical setting the same surgeon performed all the knots in multiple sessions (4 specimens each) but without standardizing the tension applied to each knot when tying it. Leakage from the proximal end of the artery was a limitation encountered by Rijkenhuinzen et al [15] during leak testing. In our study we eliminated this limitation by the method used to connect the intravenous catheter with the artery. In fact by using the latex tubing on the jaws of the mosquito forceps and by maintaining the trocar inside the canula we produced a leak proof system. In our study it was not possible to compare the sliding knot with ligating staples device and energy-based vessel sealing in terms of time spent, and this could be a starting point for a possible future investigations.

## Conclusions

Based on our results we can conclude that when ligating mesenteric vessels in horses, monofilament suture material is preferred to multifilament suture material, and that sliding knots are comparable to ligatures closed with surgeon’s knots in providing hemostasis based on bursting pressures, and are faster to perform.

## Competing interests

Authors declare no conflict of interest

## Authors' contributions

All authors contributed equally to study design, data collection, data analysis and manuscript writing and reviewing

## Declarations

This article has been published as part of *BMC Veterinary Research* Volume 10 Supplement 1, 2014: Selected articles from the Eleventh International Equine Colic Research Symposium. The full contents of the supplement are available online at http://www.biomedcentral.com/bmcvetres/supplements/10/S1. Publication of this supplement has been funded by The British Equine Veterinary Association.
